# Protective Effects of Human Pericyte-like Adipose-Derived Mesenchymal Stem Cells on Human Retinal Endothelial Cells in an In Vitro Model of Diabetic Retinopathy: Evidence for Autologous Cell Therapy

**DOI:** 10.3390/ijms24020913

**Published:** 2023-01-04

**Authors:** Gabriella Lupo, Aleksandra Agafonova, Alessia Cosentino, Giovanni Giurdanella, Giuliana Mannino, Debora Lo Furno, Ivana Roberta Romano, Rosario Giuffrida, Floriana D’Angeli, Carmelina Daniela Anfuso

**Affiliations:** 1 Department of Biomedical and Biotechnological Sciences, Section of Medical Biochemistry, School of Medicine, University of Catania, 95123 Catania, Italy; 2 Faculty of Medicine and Surgery, University of Enna “Kore”, 94100 Enna, Italy; 3 Department of Chemical, Biological, Pharmaceutical and Environmental Sciences, University of Messina, 98122 Messina, Italy; 4 Department of Biomedical and Biotechnological Sciences, Section of Physiology, School of Medicine, University of Catania, 95123 Catania, Italy; 5 Department of Human Sciences and Quality of Life Promotion, San Raffaele Roma Open University, 00166 Rome, Italy

**Keywords:** adipose mesenchymal stem cells, pericyte-like differentiation, retinal endothelial cells, blood–retinal barrier, diabetic retinopathy, hyperglycemia, inflammation, cell-based therapy

## Abstract

Diabetic retinopathy (DR) is characterized by morphologic and metabolic alterations in endothelial cells (ECs) and pericytes (PCs) of the blood–retinal barrier (BRB). The loss of interendothelial junctions, increased vascular permeability, microaneurysms, and finally, EC detachment are the main features of DR. In this scenario, a pivotal role is played by the extensive loss of PCs. Based on previous results, the aim of this study was to assess possible beneficial effects exerted by adipose mesenchymal stem cells (ASCs) and their pericyte-like differentiated phenotype (P-ASCs) on human retinal endothelial cells (HRECs) in high glucose conditions (25 mM glucose, HG). P-ASCs were more able to preserve BRB integrity than ASCs in terms of (a) increased transendothelial electrical resistance (TEER); (b) increased expression of adherens junction and tight junction proteins (VE-cadherin and ZO-1); (c) reduction in mRNA levels of inflammatory cytokines TNF-α, IL-1β, and MMP-9; (d) reduction in the angiogenic factor VEGF and in fibrotic TGF-β1. Moreover, P-ASCs counteracted the HG-induced activation of the pro-inflammatory phospho-ERK1/2/phospho-cPLA2/COX-2 pathway. Finally, crosstalk between HRECs and ASCs or P-ASCs based on the PDGF-B/PDGFR-β axis at the mRNA level is described herein. Thus, P-ASCs might be considered valuable candidates for therapeutic approaches aimed at countering BRB disruption in DR.

## 1. Introduction

Diabetic retinopathy (DR) is one of the most common causes of acquired visual impairment in developed countries [1]. It is characterized by a chronic and underhanded progression, which might be asymptomatic for years before the occurrence of clear clinical evidence. Retinas of diabetic patients exhibit both neuronal and vascular abnormalities [2]. Vision loss is the consequence of microvascular dysfunction, which causes hemorrhages, macular edema, and pathological neovascularization [3]. Temporally, the progression of DR consists of two phases: the non-proliferative (early) and the proliferative (advanced) stage. The non-proliferative stage of DR is characterized by microvessel occlusion, vascular dilation, and tortuosity of retinal microcapillaries [4,5].

Pericyte (PC) loss from the abluminal portion of the retinal microcapillaries is an early hallmark of DR and plays a key role in the dramatic evolution of the disease, due to PC inability to replicate in the adult retina [6]. PCs are highly specialized contractile cells of mesodermal origin that regulate vascular tone and perfusion pressure, both functions carried out by smooth muscle cells (SMCs) in larger vessels. Located in the wall of capillary blood vessels, PCs are embedded within the microvascular basement membrane and envelop endothelial cells (ECs), with which they establish tight physical contact [7]. Chronic exposure to glucose, advanced glycation end-product formation, hypoxia, and thickening of the basement membrane negatively affect the functional and metabolic crosstalk between PCs and ECs. This event causes PC apoptosis, impairing PC control on EC proliferation. High vessel permeability and formation of edema direct consequences of this cellular derangement [7,8].

Hyperglycemia-induced reactive oxygen species (ROS) overproduction stimulates inflammatory processes, thus contributing to the irreversible progression of DR. Elevated levels of inflammatory cytokines such as IL-1β, IL-6, IL-8, TNF-α, and MCP-1 have been found in ocular tissues from DR patients [9,10].

Matrix metalloproteinases (MMPs) modulate the extracellular matrix (ECM) composition [11,12], playing a pivotal role in both normal and pathological tissue remodeling. Among the MMP family, MMP-9 is involved in many inflammatory diseases [13], and high levels of MMP-9 and MMP-14 have been found in vitreous samples from diabetic patients [14]. Vascular endothelial growth factor (VEGF) regulates both physiological and pathological angiogenesis. High VEGF levels have been reported in the vitreous of DR patients, where it stimulates EC proliferations, migration, microvascular permeability, and the establishment of new microvessels [15,16,17,18]. Moreover, VEGF reduces the expression of both adherens junction (AJ) proteins (VE-cadherin, β-catenin), and tight junction (TJ) proteins such as zona occludens-1 (ZO-1), occludins, and claudins, thus destabilizing cell-to-cell connections [19,20]. Platelet-derived growth factors (PDGFs) are essential for the development of the vascular system [21]. ECs specifically secrete PDGF-BB, while its receptor PDGFR-β is significantly expressed by mural cells (PCs, vascular SMCs, and vascular-associated fibroblast-like cells) [21,22]. Endothelial PDGF-BB is secreted into the basement membrane, recruiting mural cells to vessels. Sustained hyperglycemia causes the chronic imbalance in the PDGFR-β signal that impairs the physiological pericytal function. It has been proposed that PDGF-BB may be a relevant therapeutic target to protect eyes from DR [23].

Eicosanoids derive from arachidonic acid (AA), released from phospholipids by the activity of different isoforms of phospholipases. They are converted to prostaglandins (PGs) or leukotrienes (LTs) by cyclooxygenases (COXs) and 5-lipoxygenase, respectively. In diabetic rat models, phospholipases A2 (PLA2) are activated, with the subsequent breakdown of the BRB [24], whereas the systemic inhibition of lipoprotein-associated PLA2 efficaciously prevents hyperglycemia-induced BRB dysfunction in rats [25]. In an in vitro model of DR, where human retinal endothelial cells (HRECs) were co-cultured with PCs, the inhibition of PLA2 reduced PGE2 and VEGF production. High glucose (HG) conditions increased HRECs and reduced PC number in co-cultures, with all effects reversed by HREC transfection with small interfering RNA targeted to PLA2. Moreover, in microvessels isolated from the retina of diabetic rats, PLA2 and COX-2 protein expression levels were significantly increased. High levels of VEGF, ICAM-1, and TNF-α were significantly reduced by PLA2 specific inhibition, thus indicating that PLA2 upregulation could be an early step in establishing a glucose-damaged BRB, probably upstream of VEGF [26]. It has also been shown that HG-induced toxicity in HRECs is mediated by the activation of the ERK1/2/PLA2 axis, where the phosphorylation/activation of cytosolic PLA2 is the major limiting step in the production of arachidonic acid and the downstream lipid mediators of inflammation [27].

At present, to mitigate hyperglycemia-induced BRB damage, medical options include laser treatment, vitrectomy, and pharmacotherapy; unfortunately, pericyte loss is virtually irreversible [6,28]. Current pharmacological treatment includes anti-VEGF drugs and corticosteroids, which are administered intravitreally. Recent clinical trials based on intraocular administration of anti-VEGF treatments have prompted researchers to re-evaluate their side effects [29]. Although these therapies have improved prognosis, repeated administrations are required, and endophthalmitis or infections are sometimes reported [17].

For these reasons, studies in vitro and on animal models have explored stem-cell-based therapies. However, data demonstrating long-term efficacy and safety are still pending [30]. The use of mesenchymal stem cells (MSCs) has been tested with encouraging outcomes for optic neuropathy, age-related macular degeneration, retinitis pigmentosa, DR, and glaucoma. Many studies have been centered on MSCs derived from bone marrow, umbilical cord, conjunctiva, and from adipose tissue [31,32]. In particular, it has been demonstrated that adipose-derived MSCs (ASCs) were able to reverse the HG-induced reduction in physiological angiogenesis in human retinal microvascular ECs, and exert a therapeutic role in DR through their pericyte-like functions, cell-to-cell contact, and the secretion of factors and cytokines [33].

Previous data from experiments on HG-cultured pericyte-like differentiated ASCs (P-ASCs) showed significant increases in mRNA expression levels of anti-inflammatory cytokines, and significant reductions in ROS production and pro-inflammatory cytokines IL-1β and TNF-α, indicating that these cells could represent a valuable tool for the treatment of retinal damage occurring in diabetic patients. For these reasons, and taking into account our previous data [34], we set out to further investigate whether the biochemical behavior of HRECs exposed to HG concentrations could benefit from the presence of ASCs or P-ASCs. In particular, in vitro strategies of co-cultures were designed to test the hypothesis that ASCs can improve the status of the endothelium, and counteract the deleterious effects of the high glucose concentrations that characterize the onset of DR.

## 2. Results

### 2.1. Effect of ASCs and P-ASCs on Transendothelial Electrical Resistance Impaired by HG

As mentioned, the BRB is significantly affected by HG conditions, which disrupt the endothelial–pericyte barrier, with the disastrous consequence of edema and leakage. Transendothelial electrical resistance (TEER) usually provides a good in vitro estimation of BRB integrity [35].

**Figure 1:**
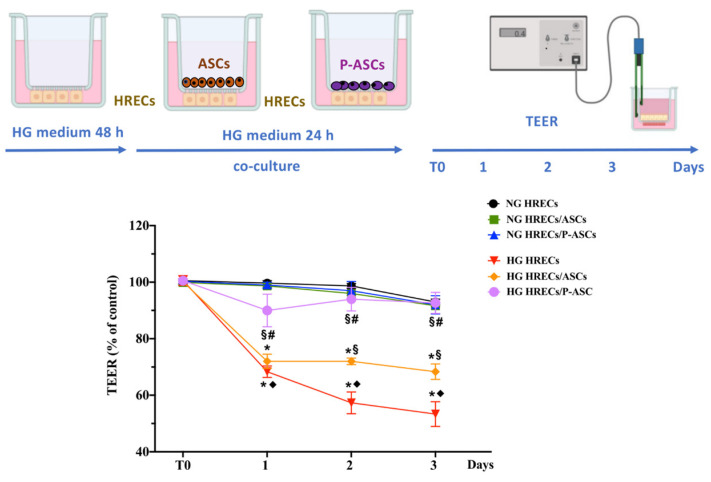
Evaluation of blood–retinal barrier integrity by transendothelial electrical resistance (TEER) in monocultures of human retinal endothelial cells (HRECs) and in co-cultures of HRECs with adipose-derived mesenchymal stem cells (ASCs) or pericyte-like differentiated ASCs (P-ASCs). In the upper part of the figure, TEER measurement procedures are illustrated. HRECs were first seeded in the underside of transwell inserts. In some samples, glucose was added to mimic hyperglycemic (HG) conditions. After 48 h, ASCs or P-ASCs were also seeded on the topside. TEER values were initially measured after 24 h of co-culture (T0), and for the following 3 days. In some other samples, TEER measurements were carried out on corresponding cultures maintained in normal glucose (NG) conditions. TEER values were also measured in cultures of HRECs alone, in NG or HG conditions, at corresponding time points. The results obtained are reported in the graph in the lower part of the figure. For each sample, data are expressed as percentage changes with respect to the value measured at T0, assumed as a control. As was expected, no significant variations were observed in samples kept in NG conditions. Instead, different changes were found for the various HG samples: the most evident decreases were exhibited by HG HRECs; except for day 1, lower decreases were noticeable when HRECs were co-cultured with ASCs; minimal decreases were observed when P-ASCs were also present. In fact, in the last case, no significant variations were revealed at day 2 and 3, compared with NG conditions. Values are expressed as the mean ± standard error of the mean (SEM) of results from three independent experiments, with four parallel samples per group in each experiment. * *p* < 0.05 vs. T0 (starting point of measurements); ♦ *p* < 0.05 vs. NG respective controls (CTRL); § *p* < 0.05 vs. respective HG; # *p* < 0.05 vs. HRECs/ASCs-conditioned media. One-way ANOVA, followed by Tukey’s test. Created with BioRender.com.

Figure 1 shows the changes in TEER values when HRECs were co-cultured with ASCs or P-ASCs, both in NG and HG conditions. As expected, in HRECs alone, TEER values in HG conditions showed a progressive decrease of almost 31%, 42%, and 43% at 1, 2, and 3 days of culture, respectively, when compared with NG conditions.

Higher TEER values were measured when HRECs were co-cultured with ASCs in HG conditions; in this case, compared with HG-HRECs alone, TEER values showed significant increases at 2 and 3 days, by almost 26% and 28%, respectively. Much more evident effects were obtained when HRECs were co-cultured with P-ASCs in HG conditions; compared with HG-treated HRECs, TEER values were significantly higher already at day 1 (by about 32%), and even higher at day 2 and 3 (65% and 73%, respectively). It is reasonable to conclude that P-ASCs were able to significantly prevent any HG-induced TEER decreases at all time points considered. It is worth noting that, except for the small decrease detected at day 1, TEER values were almost comparable to those measured in the corresponding co-cultures in NG conditions. From a quantitative point of view, P-ASCs were significantly more efficacious than ASCs at day 1, 2, and 3 of co-culture (1.25-, 1.3-, and 1.35-fold better, respectively). In control experiments, incubation with 25 mM mannitol did not change TEER values, both in mono- and in co-cultures, as compared with controls in NG conditions.

We can thus conclude that, compared with undifferentiated ASCs, a substantial protective effect is exerted by P-ASCs, showing a more stabilizing effect on the integrity on this in vitro model of the BRB.

### 2.2. ASC and P-ASC Effects on Junction Protein Expression

**Figure 2:**
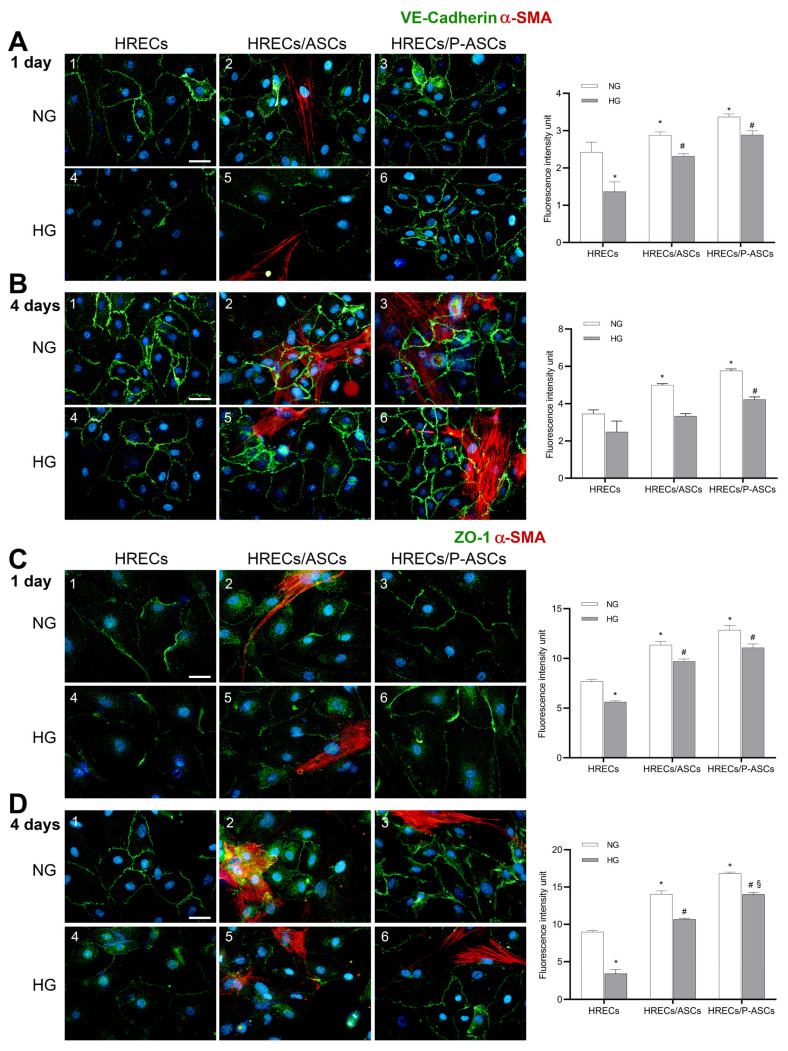
Double-labeling immunofluorescence experiments in co-cultures of human retinal endothelial cells (HRECs) and human adipose-derived mesenchymal stem cells (ASCs) or pericyte-like ASCs (P-ASCs). Micrographs obtained after 1 day (**A**–**C**) and 4 days (**B**–**D**) of mono- or co-culture, in NG or in HG. Photos A1, B1, C1, and D1 refer to HREC monocultures, taken as a reference: only VE-cadherin (**A**,**B**) or ZO-1 (**B**–**D**) immunostainings are present. In both control NG-HRECs, at day 4 (B-1 and D-1), the immunofluorescence signals were higher than at day 1 (A-1 and C-1), revealing the greater time-dependent expressions of both junction proteins and confirming the characteristic intercellular contacts, typical of blood–retinal barrier cells. In NG, the fluorescent signals were significantly stronger when HRECs were in direct mixed cultures with ASCs (A2, B2, C2, and D2) and, even more, with P-ASCs (A3, B3, C3, and D3) for both proteins at the two time points. The fluorescence related to both VE-cadherin and ZO-1 reduced significantly in HG incubation (A4, B4, C4, and D4). However, strong recoveries were observed in HRECs conditioned by ASCs (VE-cadherin, A5 and B5; ZO-1, C5 and D5) and, more significantly, by P-ASCs (VE-cadherin, A6 and B6; ZO-1, C6 and D6). No α-SMA immunoreactivity is noticeable in A1-2, B1-2, C1-2, and D1-2, which refer to HRECs in monoculture. DAPI staining of cell nuclei is indicated by blue fluorescence. Overall, these results indicate that, even in HG concentrations, the presence of P-ASCs is able to preserve the integrity of junction proteins. Scale bar: 50 µm. Histograms on the right side of each panel refer to quantification of fluorescence intensity. Values are expressed as the mean ± SEM of results from three independent experiments, with four parallel samples per group in each experiment. * *p* < 0.05 vs. HRECs in NG; # *p* < 0.05 vs. HRECs in HG; § *p* < 0.05 HRECs/P-ASCs in HG vs. HRECs/ASCs in HG.

Diabetes causes a marked reduction in the levels of VE-cadherin [36] and of tight junction ZO-1 in retinal microcapillaries [37]. We tested the hypothesis that HG could affect the expression of the above-mentioned proteins in our BRB in vitro model, and that the presence of ASCs or P-ASCs could modulate their expression. Immunofluorescence expression levels of VE-cadherin and ZO-1 in control and HG-treated HRECs, either alone or in mixed co-culture with ASCs or P-ASCs, at 1 day and 4 days of treatment, are illustrated in Figure 2. Compared with HRECs alone, VE-cadherin expression at both 1 day (panel A-1) and 4 days (panel B-1) increased in the presence of ASCs already in NG conditions (by 19 and 45%, panels A-2 and B-2, respectively) and, even more, in the presence of P-ASCs (by 25 and 67%, panels A-3 and B-3, respectively).

In HG conditions, fluorescence levels in HRECs alone were reduced by 44 and 28% at 1 day (panel A-4) and 4 days (panel B-4) of incubation, respectively, indicating a weakening of the endothelial barrier. In the same HG conditions, the co-incubation of HRECs with P-ASCs showed significantly higher VE-cadherin levels at 1 day (by about 2.1-fold, panel A-6) and at 4 days (about 1.7-fold, panel B-6). Smaller increases were observed by the presence of ASC: 1.7- (panel A-5) and 1.3-fold (panel B-5) at day 1 and day 4, respectively.

The same trend was observed for ZO-1 immunoexpression, both at 1 day (panel C) and 4 days (panel D), both in NG and HG conditions. Again, the most evident effects were induced by P-ASC treatment, showing increases of 1.96-fold at 1 day (panel C-6) and a 4.1-fold increase at 4 days (panel D-6). On the whole, P-ASC effects were more pronounced than undifferentiated ASCs. 

These data strongly support a beneficial effect of P-ASCs in preserving the anatomical and physiological role of the BRB in vitro.

### 2.3. ASC and P-ASC Modulation of mRNA Levels of Pro-Inflammatory Cytokines and VEGF in HRECs in NG and HG Conditions

**Figure 3:**
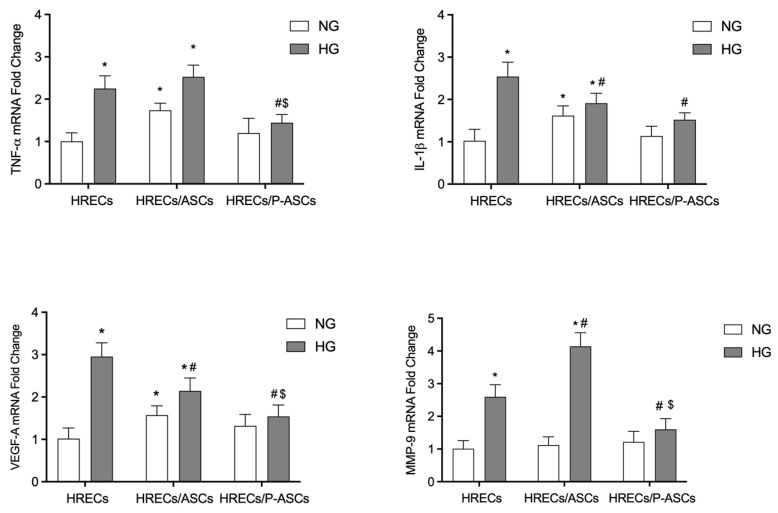
TNF-α, IL-1β, and VEGF mRNA levels in cultures of human retinal endothelial cells (HRECs) alone and co-cultured with human adipose-derived mesenchymal stem cells (ASCs) or pericyte-like ASCs (P-ASCs). TNF-α, IL-1β, and VEGF mRNA levels in HRECs were measured by qRT-PCR from samples that were cultured either in normal glucose (NG) or high glucose (HG) conditions. mRNA levels of each group were normalized to the housekeeping reference gene ribosomal 18S RNA. In the histograms, values are expressed as the fold change in those detected in HRECs cultured in NG conditions. Data indicate a modulation of inflammation genes in contrast to an HG-induced pro-inflammatory HREC phenotype. At the same time, decreases in pro-angiogenic factors were found. More evident effects were observed when HRECs were co-cultured with P-ASCs. Each value represents mean ± SEM obtained from three independent experiments. * *p* < 0.05 vs. HRECs in NG; # *p* < 0.05 vs. HRECs in HG; $ *p* < 0.05 HRECs/P-ASCs in HG vs. HRECs/ASCs in HG.

A considerable decrease in mRNA levels of pro-inflammatory cytokines and VEGF has recently been shown in cultures of P-ASCs, rather than ASCs, in response to elevated glucose concentrations [34]. We extended the study on HRECs to include co-culturing with ASCs or P-ASCs. In particular, TNF-α, IL-1β, angiogenic factor VEGF, and metalloproteinase MMP-9 mRNAs levels were investigated at 4 days of incubation, under NG and HG concentrations (Figure 3).

In HG-treated HREC monocultures, TNF-α mRNA levels increased by 2.2-fold compared with cells maintained in NG conditions. In the same conditions, however, the presence of co-cultured P-ASCs significantly reduced endothelial mRNA levels (36% reduction), whereas in HRECs co-cultured with ASCs, a slight increase was observed, showing that ASCs favor a pro-inflammatory state in HRECs both in the presence and absence of glucose. In particular, ASC-induced upregulation of mRNA levels for this cytokine was 1.7-fold in the absence, and 1.1-fold in the presence, of HG, compared with the respective HREC monocultures.

IL-1β mRNA levels significantly increased in HRECs in hyperglycemic conditions (by 2.5-fold). The presence of ASCs in NG co-cultures caused an increase in its level by 1.6-fold, whereas a reduction was observed in HG conditions (by 1.4-fold). Interestingly, in HG conditions, the presence of P-ASCs induced a significant reduction in mRNA expression (by 40%), whereas no significant differences were appreciable in NG conditions. In additional experiments, mRNA expression levels of VEGF and MMP-9 were evaluated. As expected, VEGF values increased in HG-treated HRECs by 2.9-fold. The presence of ASCs caused an increase in the angiogenic factor transcription levels by 1.5-fold, whereas a reduction of 1.4-fold was observed in HG conditions. When HRECs were co-cultured with P-ASCs, no evident effects were noted in NG conditions, whereas a strong reduction was observed for VEGF mRNA levels by almost 50%. Since MMP-9 is involved in various mechanisms of DR, such as BRB breakdown, inflammation, neovascularization, and cell apoptosis [38], we also evaluated its mRNA levels in HRECs in various conditions. In NG medium, neither the presence of ASCs nor that of P-ASCs significantly modified the expression levels of the endopeptidase. In HG conditions, HREC monoculture showed an increase of approximately 2.6-fold. By co-culturing HRECs with ASCs, MMP-9 mRNA levels were exacerbated (by 1.53-fold), unlike co-cultures with P-ASCs, in which a reduction of 42% was detected. This P-ASC-induced reduction was 62% more pronounced when compared with undifferentiated ASCs.

Overall, these data indicate a modulation of inflammation genes in contrast to the state of the pro-inflammatory HREC phenotype. At the same time, decreases in pro-angiogenic factors were noted.

### 2.4. ASC and P-ASC Modulation of TGF-β1-mRNA Levels in HRECs in NG and HG Conditions

**Figure 4:**
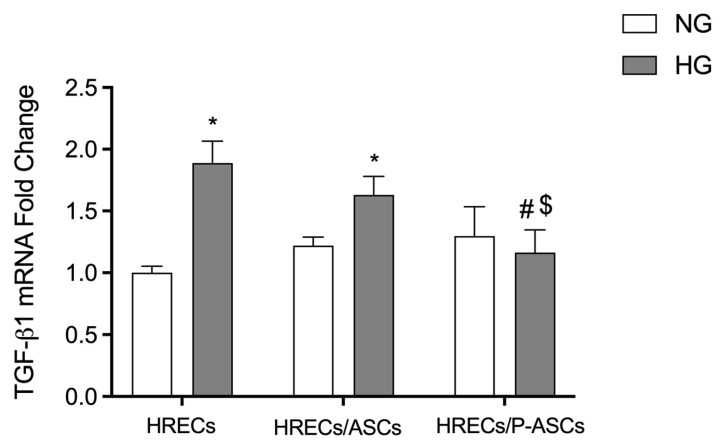
TGF-β1 mRNA levels in cultures of human retinal endothelial cells (HRECs) alone and co-cultured with human adipose-derived mesenchymal stem cells (ASCs) or pericyte-like ASCs (P-ASCs). TGF-β1 mRNA levels were measured in HRECs by qRT-PCR from samples that were cultured either in normal glucose (NG) or high glucose (HG) conditions. mRNA levels of each group were normalized to the housekeeping reference gene ribosomal 18S RNA. In the histograms, values are expressed as the fold change in those detected in HRECs cultured in NG conditions. A significant reduction in HG-induced increased TGF-β1 mRNA levels can be found when HRECs were co-cultured with ASCs and, especially, P-ASCs. Each value represents mean ± SEM obtained from three independent experiments. * *p* < 0.05 vs. HRECs in NG; # *p* < 0.05 vs. HRECs in HG; $ *p* < 0.05 HRECs/P-ASCs in HG vs. HRECs/ASCs in HG.

Since TGF-β signaling is implicated in several ocular pathologies [39], and TGF-β has been considered as a potential biomarker in DR [40], their mRNA levels were measured by quantitative RT-PCR analyses in cultures of HRECs alone or co-cultured with ASCs or P-ASCs, both in NG and in HG conditions, at 4 days of culture (Figure 4). In HG conditions, TGF-β1 mRNA showed an 88% significant increase in cultures of HRECs alone. The presence in co-cultures of ASCs, and especially P-ASCs, induced significant decreases of 1.1- and 1.6-fold, respectively. These data confirm, once again, a role of ASCs and, even more, P-ASCs in modulating HREC fibrotic cytokine levels in HG conditions. 

### 2.5. ASC and P-ASC Modulation of the ERK1/2/cPLA2/COX-2 Axis in HRECs in NG and HG Conditions

**Figure 5:**
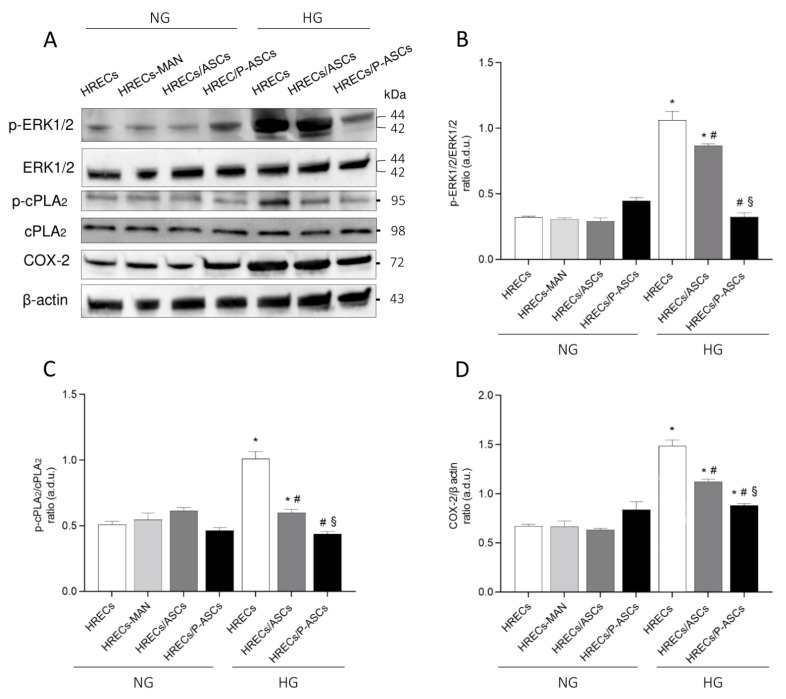
Evaluation of the ERK1/2-cPLA2-COX-2 axis in cultures of human retinal endothelial cells (HRECs) alone and co-cultured with human adipose-derived mesenchymal stem cells (ASCs) or pericyte-like ASCs (P-ASCs). Data were gathered by Western blot analysis from samples that were cultured either in normal glucose (NG) or high glucose (HG) conditions. Immunoblot analyses were performed in HREC populations using specific antibodies against p-ERK 1/2 and total ERK 1/2, activated (phosphorylated, p-cPLA2) and total cPLA2, and COX-2 proteins (panel **A**). ß actin was used to verify the equal loading of 30 μg protein per lane. Image J software was used to carry out densitometric analysis of the immunoblots, indicating protein quantification of each band (in arbitrary densitometry units, a.d.u.). Quantitative analyses of the phosphorylation rate of ERK1/2 (i.e., ratio of p-ERK1/2/ERK1/2) and cPLA2 (i.e., ratio of p-cPLA2/cPLA2) are indicated in panels **B** and **C**, respectively. Quantitative analysis of COX-2 protein was normalized to ß actin (panel **D**). No significant differences were noted among the different samples when kept in NG conditions. In HG conditions, HREC activation of the ERK1/2-cPLA2-COX-2 axis was attenuated by the presence of ASCs and, especially, P-ASCs. Each value represents mean ± SEM obtained from three independent experiments. * *p* < 0.05 vs. HRECs in NG; # *p* < 0.05 vs. HRECs in HG; § *p* < 0.05 HRECs/P-ASCs in HG vs. HRECs/ASCs in HG.

Current evidence denotes DR as a chronic inflammatory disease, and that the role of inflammation in DR is pivotal for its pathogenesis and progression [41]. In this regard, phospholipase A2 (PLA2) plays an important role in cellular injury as it mediates the inflammatory processes through the mobilization of arachidonic acid (AA) from membrane phospholipids, which is converted to eicosanoids [42]. Previous data showed an important role of PLA2 enzymes (cytosolic, cPLA2, and calcium-independent iPLA2) in glucose-induced BRB breakdown and in pericyte loss, upregulating cyclooxygenase-2 (COX-2), prostaglandin synthesis, VEGF-A, and TNF-α in vitro [8,26]. Moreover, cPLA2 activity is enhanced following phosphorylation of Ser-505 in the catalytic domain by ERK1/2, a mitogen-activated protein kinase (MAPK), and Ser-727 by other MAPKs [43,44]. Based on these assumptions, we tested the possible effects of ASCs and P-ASCs in the modulation of the expression and/or activation of the ERK1/2/cPLA2/COX-2 axis in HG-stimulated HRECs. Data obtained are summarized in Figure 5. Panel A shows immunoblots for p-ERK1/2, total ERK1/2, p-cPLA2, total cPLA2, and COX-2 proteins in lysates from HREC monocultures, as well as from HRECs co-cultured with ASCs or P-ASCs, either in NG or HG conditions. Mannitol was used as an osmotic control for HG treatment. In panel B, the quantitative densitometric analyses of bands for the phosphorylation rate of ERK1/2, indicated by the ratio p-ERK1/2/total ERK1/2, are reported. It can be easily noticed that no dramatic differences were detectable in NG conditions for all samples tested. On the other hand, HG treatment caused a significant increase in phospho-ERK1/2/total ERK1/2 ratio levels of about 3.5-fold in HREC monocultures (this increase was slightly lower), and by 2.8-fold in HRECs co-cultured with ASCs. Conversely, a significant decrease in activation of phosphorylated forms (by 70%, p-ERK1/2/total ERK1/2 ratio) was found in co-cultures with P-ASCs. No appreciable differences were noticed for total proteins in all experimental conditions.

In panel C, quantitative analysis of Western blots for the phosphorylation rate of cPLA2, indicated by the ratio p-cPLA2/cPLA2, shows that the active and phosphorylated forms of cPLA2 increased significantly in HRECs exposed to HG conditions (by 2-fold, p-cPLA2/cPLA2 ratio), in comparison to control cells. It should be noted that ASCs and, even more, P-ASCs in HG restored p-cPLA2/cPLA2 ratios to approximately control values.

In panel D, the quantification of immunoblot bands for COX-2 expression levels are reported, showing an analogous trend in protein content. In HREC monocultures, HG enhanced COX-2 expression by 2.24-fold, whereas in co-cultures with ASCs or P-ASCs, the protein enzyme content decreased by 24% and by 40%, respectively. These data indicate, again, a significant effect of P-ASCs to modulate the inflammatory response of HRECs to HG, through the deactivation of enzymes involved in the prostanoid synthesis pathway. 

### 2.6. ASC and P-ASC Modulation of PDGF-B/PDGFR-β Axis Crosstalk in HRECs in NG and HG Conditions

**Figure 6:**
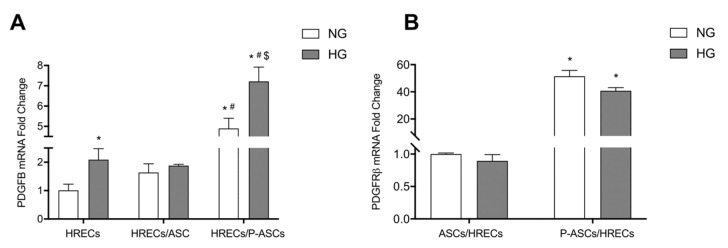
Evaluation of the PDGF-B/PDGFR-β axis in cultures of human retinal endothelial cells (HRECs) alone and co-cultured with human adipose-derived mesenchymal stem cells (ASCs) or pericyte-like ASCs (P-ASCs). By qRT-PCR, PDGF-B RNA levels were measured from HRECs (panel **A**) and PDGFR-β mRNA levels were measured from ASCs or P-ASCs (panel **B**). Cell samples that were cultured either in normal glucose (NG) or high glucose (HG) conditions were evaluated. mRNA levels of each group were normalized to the housekeeping reference gene ribosomal 18S RNA. Although the PDGF-B RNA levels were increased in cultures of HREC alone by glucose addition, much more evident increases were obtained when P-ASCs were also present, both in NG and HG conditions. On the other hand, when detecting PDGFR-β RNA levels in ASCs or P-ASCs, basal values observed in ASCs were greatly increased in P-ASCs, both in NG and HG conditions. Data indicate that enhanced pericyte recruitment would occur if P-ASCs are present. Each value represents mean ± SEM obtained from three independent experiments. Panel **A**: * *p* < 0.05 vs. HRECs in NG; # *p* < 0.05 vs. HRECs in HG; $ *p* < 0.05 HRECs/P-ASCs in HG vs. HRECs/ASCs in HG. Panel **B**: * *p* < 0.05 vs. the corresponding co-culture in NG.

The PDGF-B/PDGFR-β signaling pathway plays a pivotal role in pericyte recruitment during new vessel formation [45]. PDGF-B is the predominant isoform of PDGF in the ocular system, and is primarily expressed by vascular endothelial cells [46]; PDGFR-β is commonly expressed in PCs (in addition to SMCs, ECs, and retinal pigmented epithelium) [47]. Thus, we investigated the possible effects exerted by ASCs and P-ASCs on the overall modulation of this growth factor in HG-treated HRECs, and the possible PDGF-B/PDGFR-β axis mutual crosstalk. Compared with HRECs in NG conditions, PDGF-B levels were increased by the presence of ASCs and, much more evidently, by P-ASCs (by about 4.9-fold). In HG conditions, HREC monocultures expressed about 2.1-fold increased PDGF-B mRNA with respect to controls (Figure 6). These increases were much higher in the presence of P-ASCs (by about 3.4-fold). On the other hand, no significant differences were evident in the presence of co-cultured ASCs when compared with the corresponding NG conditions (panel A).

These data prompted us to consider PDGFR-β mRNA levels “on the side” of ASCs and P-ASCs (panel B), based on the hypothesis that this physiological signaling pathway could be involved and modulated in our in vitro system. In co-cultures with HRECs, PDGFR-β mRNA levels of ASCs were not significantly different, either in NG or HG conditions. Differently, in both conditions, HRECs induced in P-ASCs striking increases in PDGFR-β mRNA (by about 50- and 45-fold, respectively). This finding leads us to deduce, first, that the biochemical interactions between endothelium and ASCs and their differentiated P-ASCs follow the physiological path of interlocution between ECs and PCs, based on the PDGF-B/PDGFR-β axis, and second, that this path is amplified in our condition of in vitro induced hyperglycemia. 

## 3. Discussion

The use of MSCs and their administration strategies for the treatment of ocular diseases, including DR, have been previously described [31]. In in vitro and in vivo experiments, MSC administration in animal models demonstrated positive results, mainly attributable to their paracrine activity. In clinical trials, the intravitreal injection of autologous bone marrow MSCs showed good safety profiles, with some mild adverse events [48]. More recently, ASCs were increasingly investigated for tissue repair and regeneration [49,50]. They can be easily isolated and collected and, under appropriate conditions, can differentiate into a variety of cell types, such as adipocytes, osteocytes, neural cells, vascular endothelial cells, cardiomyocytes, pancreatic cells, and hepatocytes [51,52,53,54].

The therapeutic efficacy of ASCs is mainly based on their multipotent differentiation ability and their immunomodulatory properties [55,56,57]. Our recently reported data demonstrated that the in vitro differentiated human ASCs, cultured in HG conditions, showed a high proliferation rate and migration ability with respect to undifferentiated ASCs. Moreover, significant increases in mRNA expression levels of anti-inflammatory cytokines, a reduction in ROS production, and decreases in mRNA levels of pro-inflammatory cytokines and angiogenic factors were observed [34].

As observed in TEER experiments designed to evaluate the correct functionality of the BRB [58], a dramatic and progressive decrease induced by HG treatment was confirmed, indicative of BRB breakdown. However, P-ASCs, and ASCs to a significantly lesser extent, were able to counteract these detrimental effects. These results are in line with those from other in vitro models, such as human lung microvascular ECs, in which MSCs were able to partially restore protein permeability [59]. 

TEER results match those obtained by immunofluorescence for TJ and AJ proteins. In fact, it was found that, in NG conditions, VE-cadherin expression significantly increased in HREC membranes when co-cultured with ASCs and, to a greater extent, with P-ASCs. As expected, a strong decrease was found in HRECs under HG conditions, revealing a weakening of the BRB. On the contrary, the presence of P-ASCs in mixed culture caused a significant increase in VE-cadherin expression, more evident than that exerted by ASCs. These results are in line with other experimental findings, where an upregulation of endothelial VE-cadherin on the cell surface was reported, along with a reduced endothelial proliferation rate and apoptosis [60,61,62]. In our view, this P-ASC-induced strengthening effect could be attributable to their pericytal behavior. In fact, one of the activated pathways in the crosstalk between PCs and ECs, aimed at vessel stabilization, requires the activation of the angiopoietin1/Tie2 axis that improves the endothelial barrier function through the regulation of β-catenin, cytoskeletal proteins, and VE-cadherin [63,64]. It should also be noted that preserving VE-cadherin levels may have additional beneficial effects on limiting angiogenesis: VE-cadherin exerts a negative effect on EC growth through its binding to VEGFR-2 that attenuates VEGF activity [65,66,67]. The development of a novel oligonucleotide-based technology aimed at specifically increasing VE-cadherin expression in eye microvessels resulted in a reduced vascular leak in diabetic animal models, significantly strengthening pericyte coverage of retinal vessels [68]. On the other hand, ZO-1 is essential for endothelial barrier formation, spatial organization of actomyosin filaments, cell migration, and controls angiogenesis both in vitro and in vivo [69]. ZO-1 regulates the recruitment of mechanotransducers to the VE-cadherin complex [70], showing that TJs could modulate adherens junctions and endothelial function through an intricate molecular regulatory network. Hyperglycemia, oxidative stress, and inflammation are detrimental events that compromise ZO-1 expression in BRB [71]. Our data from ZO-1 immunodetection show a comparable pattern with that of VE-cadherin: HG conditions significantly reduced its expression, and co-incubation of HRECs with ASCs and, even more, with P-ASCs caused protein preservation. 

Inflammation plays a pivotal role in the pathogenesis of DR [72,73,74]. The increased production of endothelial inflammatory cytokines can generate a form of self-amplifying autocrine loop [41]. ECs are susceptible to cytokines such as IL-1β, TNF-α, and IFN-γ, which in turn stimulate the secretion of other cytokines [75]. Blocking inflammation prevents neovascularization and aberrant vessel formation [76]. We previously showed that ASCs per se show significant immunomodulatory activity even in hyperglycemic conditions, and that this trend was improved following their differentiation into P-ASCs, as indicated by the reduction in inflammation-related cytokine mRNA levels [34]. In the present study, immunomodulatory properties of ASCs and P-ASCs were tested in terms of cytokine expression by HRECs, either in NG or HG conditions. The results obtained consistently show that, compared with HREC monocultures, the simultaneous presence of P-ASCs induced a marked decrease in the pro-inflammatory cytokines TNF-α and IL-1β. These results are in line with those obtained using ASC conditioned media or after intravitreal ASC injections. 

A strong relationship has been reported between DR and both TNF-α and IL-1β pro-inflammatory cytokines [77,78,79,80]. In particular, IL-1β accelerates apoptosis of retinal capillary cells via the activation of NF-kB, and HG environments exacerbate this process [81]. As a result, the inhibition of the signaling pathway related to both cytokines attenuated retinal vascular pathologies in diabetic rodents [82,83]. Moreover, in diabetic animal models, the expression of pro-inflammatory cytokines such as IL-8, I-CAM, MCP-1, and MIP-1α was associated with increased VEGF level, which is the leading cause of vascular permeability and BRB breakdown [84,85,86,87]. In the experiments here reported, VEGF mRNA levels significantly increased in HG-treated HRECs. It is noteworthy that an appreciable reduction in the same environment condition was associated with the concomitant presence of ASCs and, to a greater extent, of P-ASCs. Overall, it is confirmed that ASCs may play an immunomodulatory paracrine role in the normalization of dysfunctional ECs in DR [88,89]. 

The sprouting angiogenesis that characterizes DR requires proteolytic activities for the degradation of the basement membrane, allowing endothelial cell migration into the matrix for the establishment of EC tubules [90]. In this context, MMP-9 increases vascular permeability by disrupting the tight junction complex [91]. MMP-9 levels are significantly elevated in plasma and retinas from patients with diabetes [92], and in the vitreous and retinas of DR patients [91,93]. In the present experiments, a significant increase in MMP-9 mRNA levels was observed in HREC cultures after glucose addition, whereas a strong reduction was observed in the presence of P-ASCs.

TGF-β1 is a multifunctional growth factor that regulate EC functions by differentially activating two types of receptors, ALK5 and ALK1, and intracellular SMAD effectors [94]. The physical contact between ECs and mesenchymal cells leads to its activation [95], and constitutive TGF-β signaling is essential to maintain retina integrity [96]. The double role of TGF-β (TGF-β “butterfly effect” or “paradox”) during tumorigenesis has often been described, as well as its related angiogenesis, raising questions about its boundary between physiological and pathological effects [96]. Transgenic model studies highlighted that the loss of TGF-β signaling components elicit irregular capillary plexus formation [97]. On the other hand, TGF-β/Smad3 pathway activation is described as fibrogenic signaling bearing the common characteristics of clinically significant fibrosis in various tissues of diabetic patients through different mechanisms [98]. Its pathological involvement is particularly relevant in HG-induced EC dysfunction as it triggers the endothelial to mesenchymal transition processes, increasing diabetic complications by stimulating ECM production [98]. In our model, we observed that the protective effect of P-ASCs on HG-stimulated HRECs is achieved by reverting the TGF-β1 hyperglycemia-induced upregulation.

In diabetes, the increased PGE2 production from COX-2 activity significantly contributes to cellular dysfunction [8,26,99]; on the other hand, COX-2 inhibition prevents glucose-induced overproduction of VEGF-A in the retina [100,101]. Furthermore, the specific inhibition of calcium-dependent cPLA2 protects HRECs against glucose-induced damages, and cPLA2 would contribute to the early HG-induced damage by the upregulation of retinal VEGF-A [102]. Moreover, the HG-induced VEGF-A upregulation is related to the cPLA2 phosphorylation, which coincides with its activation. In fact, the specific VEGF-A inhibitor Aflibercept was able to prevent the phosphorylation levels of cPLA2 and of the upstream ERK1/2, and to reduce HG-induced cell damage [27]. 

In agreement with the above-reported data, the results obtained in this work reveal that the active/phosphorylated form of cPLA2 significantly increased in HRECs exposed to HG concentrations. Total cPLA2 expression remained unchanged in all experimental conditions. As was expected, COX-2 protein levels followed the pattern of cPLA2 activation. When analyzing HG conditions, a robust increase in COX-2 expression was evident in HREC monocultures. These increases were less pronounced when ASCs were also present, and almost equivalent to NG conditions when HRECs were co-cultured with P-ASCs. In our opinion, COX-2 increases coincide with the activation of its enzymatic activity, aimed at metabolizing the AA produced by cPLA2 in prostanoids. 

Similar to previous results, the activation of the cPLA2/COX-2/PGE2 that occurs immediately after exposure of HRECs to elevated glucose concentrations is closely dependent on the activation of MAPK-ERK1/2 [8,26]. In infected INS-1 cells, and in glioma-conditioned-medium-stimulated ECs, PD98059, a specific MEK1 kinase inhibitor, significantly reduced cPLA2 enzyme activity [103,104,105]. HG treatment caused a significant increase in phospho-ERK1/2 levels, however, when HRECs were co-cultured with P-ASCs, a significant decrease in phosphorylated forms was found, in line with the trend of cPLA2 activation.

The recruitment of PCs in the developing retina and cerebral vessels occurs through the PDGF-B/PDGFR-β axis, and it is a key event for the proper formation of the BRB and the BBB [106,107]. Park et al. demonstrated that impaired PDGF-B/PDGFR-β signaling in growing retinal vessels shows various features common to DR [45]. In a mouse model of focal cerebral ischemia, the PDGFR-β signaling enhancement in PCs can improve BBB integrity and minimize cerebral edema. Synergistic mechanisms of PDGFR-β and TGF-β signaling on the restoration of BBB integrity and functions have been described [108]. Because of the critical role of PDGF-BB/PDGFR-β signaling in preserving PC coverage and vascular stability, we explored potential changes in this biochemical axis in the reciprocal dialogue between HRECs and ASCs or P-ASCs under HG-conditions.

**Figure 7:**
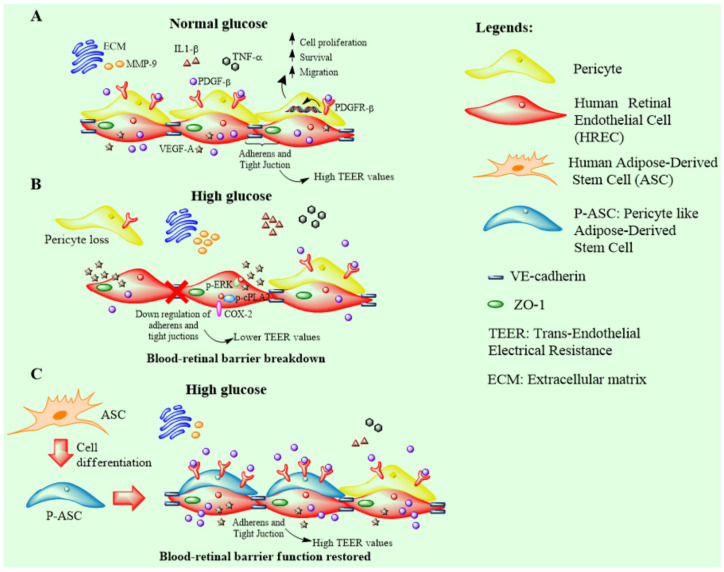
Schematic diagram of blood–retinal barrier (BRB) integrity restored (preserved) by the treatment with pericyte-like differentiated adipose-derived mesenchymal stem cells (P-ASCs) in the context of diabetic retinopathy. (**A**) In normal glucose conditions, human retinal endothelial cells (HRECs) tightly adhere to each other by both adherens (VE-cadherin) and tight junctions (zonula occludens-1; ZO-1), providing protection to retinal structures. The presence of cell junctions is responsible for high transendothelial electrical resistance (TEER) values, which are indicative of BRB integrity. In a health context, the release of inflammatory cytokines (i.e., interleukin-1β (IL-1β) and tumor necrosis factor-α (TNF-α)), pro-angiogenic factor, vascular endothelial growth factor-A (VEGF-A), and matrix metalloproteinase-9 (MMP-9) is limited to satisfying homeostatic needs only. HRECs produce and release the platelet-derived growth factor-β (PDGF-β), which is essential for the recruitment of pericytes to vessels. This molecule interacts with the platelet-derived growth factor receptor-β (PDGFR-β), located on the pericyte surface, triggering an intracellular signaling cascade that ensures their survival, proliferation, and migration. (**B**) In diabetic retinopathy, high glucose concentrations cause BRB breakdown, characterized by a series of events involving endothelial cells, pericytes, and the extracellular environment. In HRECs, high glucose levels induce the activation of the p-ERK1/2/p-cPLA2/COX-2 axis associated with the inflammatory state. Moreover, the connections between endothelial cells become weaker because of the downregulation of both adherens and tight junctions. This condition, revealed by lower TEER values, makes the BRB more accessible to external insults. Endothelial cells also overexpress VEGF-A, which leads to increased angiogenesis correlated to inflammation. Furthermore, the excess of glucose causes a reduction in PDGF-β release from HRECs, and a concomitant decrease in PDGFR-β expression on pericytes. As a result, pericyte population density decreases, leaving endothelial cells unprotected. At the extracellular level, hyperglycemia provokes an inflammatory state supported by the release of IL-1β and TNF-α, in addition to the increased release of MMP-9, responsible for the remodeling of the extracellular matrix. (**C**) Intraocular administration of P-ASC might result in beneficial effects, since these cells could act as substitutes for lost pericytes, thus limiting hyperglycemia-induced deleterious effects and possibly restoring BRB integrity.

An appreciable increase in PDGF-B mRNA was found in HRECs when exposed to HG concentrations. This increase was substantially augmented by the presence of P-ASCs. At the same time, when considering the PDGFR-β mRNA levels in P-ASCs, an impressive increase was revealed. In our opinion, the physiological crosstalk between ECs and PCs would remain efficient, even at HG concentration, if P-ASCs take the place of lost pericytes. Taken together, these data provide further evidence of the pericyte-like behavior of in vitro differentiated ASCs. The contact, under normal conditions, with HRECs induces the overexpression of PDGFR-β, evoked and reinforced by the upregulation of endothelial PDGF-BB production. The physiological endothelial signal, normally aimed at the recruitment of PCs during the formation of new microcapillaries, would be maintained and amplified in hyperglycemic conditions. A schematic illustration of these data and of our speculations is reported in Figure 7.

It can be concluded that P-ASCs could be considered potential substitutes for pericytes in a hyperglycemic context characterized by pericyte loss in DR (pursuing the idea of inoculating autologous P-ASCs into the diabetic eye), in an attempt to reduce retinal microvessel damage and BRB breakdown.

## 4. Materials and Methods

### 4.1. Cell Cultures

#### 4.1.1. Human Retinal Endothelial Cells (HRECs)

Primary human retinal endothelial cells (HRECs) were purchased from Innoprot (Elexalde, Derio, Spain) and cultured with endothelial cell medium (ECM) supplemented with 5% fetal bovine serum (FBS), 1% endothelial cell growth supplement (ECGS), 1% penicillin/streptomycin solution (P/S solution), all purchased from Innoprot. The nature of HRECs was confirmed by immunostaining for the von Willebrand factor (Biomedical Technologies Inc., Stoughton, MA, USA) [44]. Cells were treated when at about 70% confluence. Before all treatments, HRECs were adapted to ECM medium with 2.5% FBS for 24 h. Cells were then incubated under control conditions (normal glucose, NG) or with 25 mM glucose (high glucose, HG) in 2.5% FBS ECM medium.

#### 4.1.2. Human Adipose Stem Cells (ASCs)

Adipose tissue was harvested from four healthy female donors (32–38 years old) undergoing liposuction procedures at the Cannizzaro Hospital, Catania (Italy). The donors were non-smokers, and did not take estrogen replacement therapy. Lipoaspirate was obtained from the abdominal region after donors had signed an informed consent form for the use of lipoaspirate for experimental procedures, in accordance with the Declaration of Helsinki. The protocol was approved by the local ethics committee (Ethic committee Catania1; Authorization n. 155/2018/PO). The raw lipoaspirate (50–100 mL) was incubated for 3 h at 37 °C with an equal volume of serum-free low-glucose Dulbecco’s Modified Eagle’s Medium (DMEM; Sigma-Aldrich, Milan, Italy) containing 0.075% type I collagenase (Invitrogen, Monza, Italy). After inactivation of collagenase activity by adding an equal volume of DMEM containing 10% heat-inactivated FBS (Gibco, Monza, Italy), the digested lipoaspirate was centrifuged at 1200 rpm for 10 min. The pellets were then washed in phosphate-buffered saline (PBS; Invitrogen), filtered through a 100 µm nylon cell strainer (Falcon BD Biosciences, Milan, Italy) and the cells were plated in T75 culture flasks (Falcon BD Biosciences) with DMEM containing 10% FBS, 1% P/S solution, and 1% MSC growth supplement (ScienCell Research Laboratories, Milan, Italy). After 24 h incubation at 37 °C with 5% CO2, non-adherent cells were removed by replacing the growth medium. When reaching confluence, all cultures were expanded for 2–3 passages and plated for the subsequent procedures. Some cell samples were used to verify their MSC nature, according to procedures previously described [109]. In particular, their positivity for typical MSC markers (CD44, CD73, CD90, and CD105) was confirmed by immunocytochemistry and flow cytometry, and their negative immune response for typical hematopoietic stem cell markers (CD14, CD34, and CD45) was verified.

#### 4.1.3. Pericyte-like Differentiation of ASCs (P-ASCs)

According to a previous study [110], the pericyte like-differentiation of ASCs was achieved by their growth for 3 days in a culture medium specifically designed for pericytes (PM; Innoprot) containing 2% FBS and 5 mM glucose (normal glucose, NG). As expected, in these samples, α-SMA and NG2 expression levels were significantly higher than control ASCs that were cultured in their basal medium (DMEM containing 2% FBS in NG conditions). Two culture groups were then obtained: ASCs and pericyte-like ASCs (P-ASCs), both used for performing co-cultures with HRECs.

#### 4.1.4. HRECs/ASC and HRECs/P-ASC Co-Cultures

In order to evaluate the reciprocal interactions between HRECs and ASCs or P-ASCs, two co-culture strategies were performed. In mixed direct co-cultures, HRECs were allowed to contact directly with ASCs or P-ASCs. Indirect co-cultures in multiwell plates with transwell inserts (Falcon permeable transparent PET membrane inserts for six-well plates, pore size of 0.4 µm; Falcon BD Biosciences) were set up to mimic an in vitro BRB model system. In this case, HRECs were plated on the underside of the insert and ASCs or P-ASCs on the topside [44]. For direct co-cultures, HRECs were plated on the coverslips in multiwell plates (2 × 10^4^ cells/coverslip). The following day, 25 mM glucose (HG) was added to some samples to mimic hyperglycemic conditions, whereas control cells were kept in NG conditions [34]. After 48 h in HG conditions, ASCs or P-ASCs (5 × 10^3^ cells/well) were added to each HREC sample. As a result, four co-culture groups were obtained: HRECs/ASCs and HRECs/P-ASCs in NG conditions; HRECs/ASCs and HRECs/P-ASCs in HG conditions. HRECs/ASCs were cultured in a mixed medium containing 50% ECM and 50% basal ASC growth medium, whereas a combination of 50% ECM and 50% PM was used for HRECs/P-ASCs. Additional cultures of HRECs alone (in NG and in HG conditions) were used as controls. The cells were fixed at different time points: after 1 day and 4 days in mixed direct co-cultures. At each time point, a double-labeling immunofluorescence procedure was carried out to simultaneously reveal α-SMA and VE-cadherin or ZO-1.

In transwell co-cultures, possible effects of ASCs or P-ASCs on HREC response to hyperglycemia were tested. Briefly, HRECs were first plated on the underside of the inserts at a density of 1.5 × 10^5^ cells/well. After 4 h, the inserts were turned upside down in the culture plate [44,103]. The following day, 25 mM glucose was added to HG-treated cells. Corresponding control co-cultures were kept in NG medium. After a further 2 days in culture, ASCs or P-ASCs (7.5 × 10^3^ cells/well) were added to the topside of the transwell inserts, opposite to the underside HRECs. As in mixed direct cultures, four culture groups were obtained: HRECs/ASCs and HRECs/P-ASCs in NG and HG conditions. Additionally, in this case, HRECs/ASCs were cultured in a mixed medium containing 50% ECM and 50% basal ASC growth medium. Instead, a combination of 50% ECM-50% PM was used for HRECs/P-ASCs. In addition, cultures of HRECs alone (in NG and in HG conditions), seeded on the underside of the transwell inserts, were used as controls. These indirect co-cultures were used to evaluate the transendothelial electrical resistance (TEER) and perform immunoblot analyses and quantitative RT-PCR (qRT-PCR).

### 4.2. Immunofluorescence Assays

In mixed direct co-cultures, immunocytochemical staining was carried out following the same procedures previously described [110]. Cells were washed with PBS and fixed at −20 °C with acetone (15 min) and methanol (20 min). In the following step, cells were incubated for 30 min with a 5% solution of normal goat serum (Sigma-Aldrich) in PBS containing 0.1% Triton (Sigma-Aldrich). They were then exposed overnight at 4 °C to primary antibodies: mouse anti α-SMA (1:200; M0851, Dako, Milan, Italy); rabbit anti VE-cadherin (1:400; D87F2, Cell Signaling Technology, Danvers, MA, USA); rabbit anti ZO-1 (1:100; 61–7300, Invitrogen). The following day, cells were washed with PBS and incubated for 60 min at room temperature with secondary antibodies conjugated to different fluorochromes: FITC-conjugated goat anti-rabbit (1:500; ab6717, Abcam, Boston, MA, USA) and/or Cy3-conjugated goat anti-mouse (1:500; ab97035, Abcam). The specificity of immunostaining was verified in control experiments by omitting the primary antibody. Finally, DAPI staining was used to visualize cell nuclei (10 min).

Quantification of the fluorescence intensity of ZO-1 and VE-cadherin was performed using the ISO data threshold method using ImageJ analysis software (version 1.53e). Fluorescence intensity data were normalized on the total area and expressed as mean fluorescence intensity ± SEM.

### 4.3. Transendothelial Electrical Resistance (TEER) Measurement

TEER was measured with the Millicell-ERS system (MERS 000 01; Millipore, AG, Volketswil, Switzerland), as previously described [58,103,111]. In order to achieve optimal HREC confluent monolayers or co-cultures, in NG or HG media, TEER value measurements were carried out in transwells daily for 4 days. Values from empty transwell inserts were considered as blank wells. Experimental values were expressed as Ω/cm^2^ and were calculated by the formula: [average resistance of experimental wells—average resistance of blank wells] × 0.33 (the area of the transwell membrane). 

### 4.4. Extraction of Total mRNA and Quantitative Real-Time Reverse Transcriptase Polymerase Chain Reaction (qRT-PCR)

After 4 days of co-culture, qRT-PCRs were performed in HRECs to determine mRNA levels of VEGF-A, IL-1β, TGF- β1, TNF-α, MMP-9, PDGFB, PDFGR β, and 18S rRNA. Briefly, total cellular mRNA was extracted using QIAzol reagent (79306, QIAGEN Inc., Valencia, CA, USA) according to the manufacturer’s instructions, and dissolved in 15–20 μL of RNase-free water. The RNA concentration and purity were estimated by optical density measurements at 260 and 280 nm, respectively. Reverse transcription of mRNA (1 µg) into first-strand cDNA was accomplished using the QuantiTect Reverse Transcription Kit (205313, QIAGEN Inc.).

First, genomic DNA was eliminated by incubation at 42 °C for 2 min. Subsequently, cDNA synthesis was carried out at 42 °C for 15 min, and Quantiscript Reverse Transcriptase was inactivated by incubation at 95 °C for 3 min. Aliquots of cDNA (50 ng) were amplified in parallel reactions by employing iTaq Universal SYBR Green Supermix (1725124, Bio-Rad, Milan, Italy) in a final volume of 10 µL with 0.8 µM primers. Primers were supplied by Eurofins Genomics Germany GmbH (Ebersberg, Germany). The specific set of primers is reported in Table 1. Amplifications were carried out in a 7300 Real Time PCR System (Applied Biosystems, Thermo Fisher Scientific, Waltham, MA, USA). Negative controls were included in each assay. qRT-PCR data were analyzed by the comparative threshold cycle method (ΔΔCt). All samples were run in triplicate and normalized by the expression of the endogenous 18S rRNA gene. 

### 4.5. Immunoblot Analyses

Western blotting was used to analyze the proteins that were extracted from whole cell lysates [112]. After 4 days of indirect co-culture, cells were washed twice with PBS and harvested mechanically using a cell scraper. Then, cells were centrifuged at 1000 rpm for 5 min at 25 °C, and pellets were lysed in RIPA buffer (20188, EMD Millipore Corporation, Temecula, CA, USA) supplemented with protease and phosphatase inhibitor cocktails (Protease Inhibitor Cocktail Set III EDTA-Free, 539134, EMD Millipore Corporation; Phosphatase Inhibitor Cocktail 2, P5726, and Phosphatase Inhibitor Cocktail 3, P0044, Sigma-Aldrich, St. Louis, MO, USA). Cell lysates were centrifuged at 13,000 rpm for 20 min at 4 °C, and protein concentrations in the supernatant were determined by the BCA protein assay (BCA Protein Assay Kit; sc-202389, Santa Cruz Biotechnology, Santa Cruz, CA, USA).

Protein extracts (30 μg) were loaded on 4–20% precast polyacrylamide gel (Mini-PROTEAN^®^ TGXTM Precast Protein Gels; 4561096, Bio-Rad Laboratories, Segrate, Italy), separated by SDS-PAGE, and electrotransferred to nitrocellulose membranes (Trans-Blot Turbo Mini 0.2 μm nitrocellulose transfer packs; 1704158, Bio-Rad Laboratories). The membranes were blocked for 30 min in Odyssey blocking buffer (LI-COR Biosciences, Lincoln, NE, USA), and incubated at 4 °C overnight with the following primary antibodies: phospho-ERK 1/2 (1:1000; 9101S, Cell Signaling Technology), total ERK 1/2 (1:1000; 9102S, Cell Signaling Technology), phospho-cPLA2 (1:1000; 2831S, Cell Signaling Technology), total cPLA2 (1:500; ab239730, Abcam), COX-2 (1:500; ab88522, Abcam), and β-actin (1:1000; ab8226, Abcam) as a loading control. The membranes were then incubated for 1 h at room temperature with secondary HRP-conjugated anti-rabbit (NA934V, GE Healthcare, Bloomington, IL, USA) or anti-mouse (ab6789, Abcam) antibodies at 1:2000 dilutions. The immune complexes were detected by enhanced chemiluminescence (ECL Super-Signal West Dura Extended Duration Substrate; 34075, Thermo Fisher Scientific) using the Chemi-Doc Touch Imaging System (Bio-Rad, Hercules, CA, USA). Densitometry analyses of the blots were performed using Image J software (National Institutes of Health, Bethesda, MD, USA) [113].

### 4.6. Statistical Analysis

Experiments were performed four times (n = 4) in triplicate (biological and technical replicates). Data are reported as mean ± SEM. The different groups were compared by two-way analysis of variance (ANOVA). The non-parametric Mann–Whitney test was used for pairwise comparisons; a *p* value < 0.05 was considered a statistically significant difference between experimental and control groups. Statistical analysis and graph design were carried out by means of GraphPad Prism 9 software (GraphPad Inc., San Diego, CA, USA).

## 5. Conclusions

In conclusion, our data indicate that human P-ASCs, more than progenitor ASCs, can be considered good candidates for protecting the functional characteristics of the human BRB in DR. In in-contact co-cultures with HRECs, P-ASCs preserve TEER and VE-cadherin and ZO-1 junction protein expression, features of endothelial barrier behavior. Moreover, P-ASCs are able to reduce inflammatory cytokines such as TNF-α, IL-1β, and MMP-9, the angiogenic factor VEGF at mRNA levels, and the fibrotic TGF-β. Moreover, P-ASCs reduced the HG-induced inflammatory response in terms of the phospho-ERK1/2/phospho-cPLA2/COX-2 pathway activation. As for the P-ASCs/HRECs crosstalk, both in NG and in HG conditions, P-ASCs induced a significant increase in PDGF-B mRNA expression in HRECs, and, at the same time, HRECs determined marked increases in PDGFR-β mRNAs in ASCs and P-ASCs. In all likelihood, the mutual modulation of the PDGF-B/ PDGFR-β axis could play a pivotal role, although further investigations are needed to explore how it occurs at molecular levels. Certainly, the in vitro differentiation of ASCs into pericyte-like cells may be taken into account in the protection of the retinal endothelium in DR from the perspective of future cell therapies for the treatment for retinal vascular diseases. Finally, it should be pointed out that, compared with MSCs from other sources, ASCs are more suitable for the development of cell-based therapeutic approaches in DR as well as in many other human diseases, especially when other treatments fail. In fact, ASCs offer some important advantages: they are clearly eligible for autologous administration and can be harvested with minimal discomfort for the patient.

## Figures and Tables

**Table 1:** Set of primers used for qRT-PCR.

Gene	Sequence (5′-3′)
*VEGF-A*	Fw: ATCTTCAAGCCATCCTGTGTGC
	Rv: GAGGTTTGATCCGCATAATCTG
*IL10*	Fw: GACTTTAAGGGTTACCTGGGTTG
	Rv: TCACATGCGCCTTGATGTCTG
*IL-1β*	Fw: AGCTACGAATCTCCGACCAC
	Rv: CGTTATCCCATGTGTCGAAGAA
*TGF-β1*	Fw: CGTCTGCTGAGGCTCAAGT
	Rv: CGCCAGGAATTGTTGCTGTA
*TNF-α*	Fw: AGCCCATGTTGTAGCAAACC
	Rv: TGAGGTACAGGCCCTCTGAT
*MMP-9*	Fw: CACTGTCCACCCCTCAGAGC
	Rv: GCCAACTTGTCGGCGATAAGG
*PDGF-β*	Fw: TGA TCT CCA ACG CCT GCT
	Rv: TCA TGT TCA GGT CCA ACT CG
*PDGFR-B*	Fw: TCA GCA AGG ACA CCA TG
	Rv: CCG AGC AGG TCA GAA CGA AG

## Data Availability

No additional data are available.
